# Supplementary material for: Identification of photoperiod-induced specific miRNAs in the adrenal glands of Sunite sheep (Ovis aries)
Source: Front Vet Sci. 2022 Jul 22;9:888207. doi: 10.3389/fvets.2022.888207 (PMC9354845; doi:10.3389/fvets.2022.888207)
Supplement: Supplementary file 1 [file Data_Sheet_1.ZIP › Supplementary file/miRDeep2 file.zip!/miRDeep2 file/Know_Structure/SP42A1/oar-mir-191.pdf]

miRBase precursor : oar-mir-191
Total read count : 87276
oar-miR-191 read count : 87065
remaining reads : 211

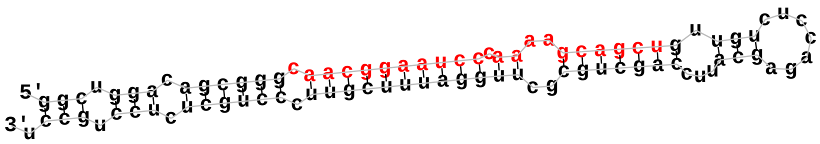

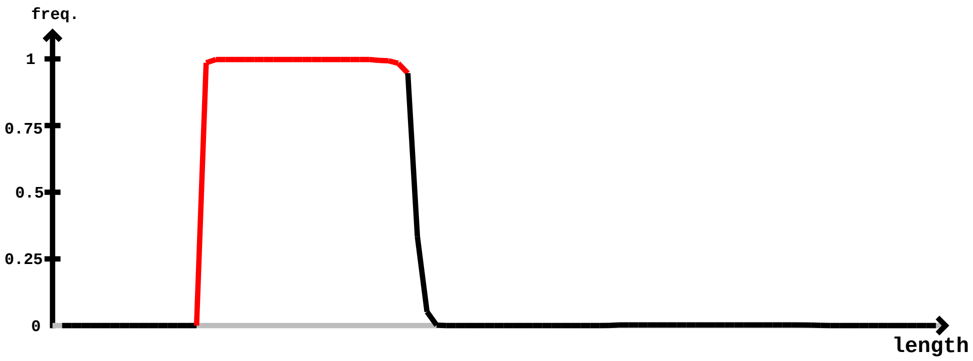


```
              oar-miR-191
5'- ggcuggacagcgggcaacggaaucccaaaagcagcuguugucuccagagcauuccagcugcgcuuggauuucguucccugcucuccugccu  -3'   exp
    (((.(((.((((((.((((((((((.((..(((((((.(((.......)))...)))))))..)))))))))))).)))))).))).)))).   reads   mm   sample
    ..............caacggaaucccaaaagc...........................................................   289     0    seq
    ..............caacggaaucccaaaagca..........................................................   127     0    seq
    ..............caacggaaucccaaaagcag.........................................................   811     0    seq
    ..............caacggaaucccaaaagcagc........................................................   3234    0    seq
    ..............caacggaaucccaaaagcagcu.......................................................   53165   0    seq
    ..............caacggaaucccaaaagcagcug......................................................   24130   0    seq
    ..............caacggaaucccaaaagcagcugu.....................................................   4104    0    seq
    ..............caacggaaucccaaaagcagcuguu....................................................   126     0    seq
    ..............caacggaaucccaaaagcagcuguug...................................................   3       0    seq
    ..............caacggaaucccaaaagcagcuguugu..................................................   1       0    seq
    ...............aacggaaucccaaaagcag.........................................................   2       0    seq
    ...............aacggaaucccaaaagcagc........................................................   16      0    seq
    ...............aacggaaucccaaaagcagcu.......................................................   329     0    seq
    ...............aacggaaucccaaaagcagcug......................................................   451     0    seq
    ...............aacggaaucccaaaagcagcugu.....................................................   273     0    seq
    ................acggaaucccaaaagcagcu.......................................................   1       0    seq
    ................acggaaucccaaaagcagcugu.....................................................   1       0    seq
    .................cggaaucccaaaagcagcu.......................................................   1       0    seq
    ..................ggaaucccaaaagcagcu.......................................................   1       0    seq
    .......................................................agcugcgcuuggauuucguucc..............   2       0    seq
    .......................................................agcugcgcuuggauuucguuccc.............   1       0    seq
    ........................................................gcugcgcuuggauuucgu.................   1       0    seq
    ........................................................gcugcgcuuggauuucguu................   1       0    seq
    ........................................................gcugcgcuuggauuucguuc...............   12      0    seq
    ........................................................gcugcgcuuggauuucguucc..............   8       0    seq
    ........................................................gcugcgcuuggauuucguuccc.............   5       0    seq
    ........................................................gcugcgcuuggauuucguucccu............   3       0    seq
    .........................................................cugcgcuuggauuucguu................   1       0    seq
    .........................................................cugcgcuuggauuucguuc...............   6       0    seq
    .........................................................cugcgcuuggauuucguucc..............   20      0    seq
    .........................................................cugcgcuuggauuucguuccc.............   91      0    seq
    .........................................................cugcgcuuggauuucguucccu............   49      0    seq
    .........................................................cugcgcuuggauuucguucccug...........   2       0    seq
    .........................................................cugcgcuuggauuucguucccugc..........   1       0    seq
    ..........................................................ugcgcuuggauuucguuccc.............   1       0    seq
```

oar-miR-191

```
ggcuggacagcgggcaacggaaucccaaaagcagcuguugucuccagagcauuccagcugcgcuuggauuucguucccugcucuccugccu
..........................................................ugcgcuuggauuucguucccu............    7          0          seq
```
